# Relationship between Classification of Fabellae and the Severity of Knee Osteoarthritis: A Relevant Study in the Chinese Population

**DOI:** 10.1111/os.13006

**Published:** 2021-12-16

**Authors:** Lei Zhang, You‐liang Wen, Chun‐ying He, Yan Zeng, Jun‐qiu Wang, Guo‐you Wang

**Affiliations:** ^1^ Department of Orthopaedics Affiliated Traditional Chinese Medicine Hospital of Southwest Medical University Luzhou China; ^2^ Center for Orthopaedic Diseases Research Affiliated Traditional Chinese Medicine Hospital of Southwest Medical University Luzhou China; ^3^ Expert Workstation in Luzhou Luzhou China; ^4^ Clinical Base of Affiliated Traditional Chinese Medicine Hospital of Southwest Medical University, Guangdong Province Medical 3D Printing Application Transformation Engineering Technology Research Center Luzhou China; ^5^ School of Rehabilitation Medicine GanNan Medical University Ganzhou China; ^6^ School of Clinical Medicine of Integrated Traditional Chinese and Western Medicine, Southwest Medical University Luzhou China; ^7^ Department of Nephrology Affiliated Traditional Chinese Medicine Hospital of Southwest Medical University Luzhou China

**Keywords:** Fabella, Knee osteoarthritis, Morphology, Classification

## Abstract

**Objective:**

To classify the fabellae and discuss the relationship between the classification of fabellae and the severity of knee osteoarthritis (KOA) in Chinese.

**Methods:**

From February 2019 to February 2020, 136 patients were measured and classified using three‐dimensional computed tomography (CT) reconstruction. According to the CT imaging characteristics, the fabellae were divided into five types: type I, a fabella on the lateral femoral condyle; type II, a fabella on the medial femoral condyle; type III, a fabella on the lateral femoral condyle and a fabella on the medial femoral condyle; type IV, two fabellae on the medial femoral condyle; and type V, two fabellae on the lateral femoral condyle. The severity of KOA was assessed on the Recht grade by magnetic resonance imaging (MRI). The data were analyzed with SPSS 24.0.

**Results:**

The classification of fabellae were correlated with KOA grades (χ^2^ = 35.026, *P* < 0.05). In terms of KOA grades, grade I and grade II were occupied most by fabellar type II (32, 72.8%); type II and other types showed significant statistical difference (*P* < 0.05). Grade I and grade II were also mainly fabellar type IV (four, 100%). Fabellar type V's biggest component was grade III and grade IV (six, 75%). Type IV and type V showed significant statistical difference (*P* < 0.05).

**Conclusion:**

The classification of fabellae were correlated with KOA grades. The type II may mean the lower KOA grades while type V may mean the higher KOA grades.

## Graphical abstract

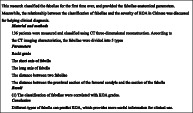

## Introduction

The fabella is a fibrocartilaginous or ossified sesamoid bone and because it often presented as a benign structure, the clinical significance of it was usually ignored^1^. However, under the mechanical stresses and loading, the fabella may act as a source of atypical knee pain in some cases, such as fabella syndrome, common fibular nerve palsy, chondromalacia, fabella dislocation, popliteal entrapment syndrome, and knee osteoarthritis (KOA)^2^, ^3^, ^4^. The physicians may recognize it as an intra‐articular loose body or an osteophyte, which could lead to delay in diagnosis and overuse of arthroscope^5^, ^6^, ^7^. So, it is really important that we investigate this issue. While there are many studies focused on the fabellar prevalence, only a few studies report on anatomical morphology of fabella^8^, ^9^.

The fabella has anatomical variations that could be located in the medial and lateral femoral condyle and is embedded in the lateral head of the gastrocnemius muscle mostly. Nevertheless, in recent years, some reports just described that the fabella was located in the knee joint behind the lateral femoral condyle^10^, ^11^, ^12^, ^13^. And while the fabella has certain anatomical variations in location and quantity, they have not been classified^14^, ^15^.

KOA is a degenerative and inflammatory joint disease which can lead to chronic pain and lower‐limb disability^16^. KOA could cause serious socio‐economic burdens, as the annual health care expenditures of KOA have been estimated at $US186bn^17^. However, KOA affects articular cartilage mostly, and the limited capacity of healing in articular cartilage indicates that it cannot be effectively repaired^18^, ^19^, ^20^. The relationship between KOA and fabellar occurrence rate has been supported. Several reviews have investigated that fabella was more common in patients with primary KOA. In their study, fabella was present in 35% of 300 patients with primary KOA and only in 15% of knees in the age‐matched control group^21^, ^22^, ^23^. Pritchett *et al*. speculated that in some way, the fabella can predict KOA and provide useful information for clinical use^24^. However, the link between the classification of fabellae and the severity of KOA remains unknown^25^.

This study aims to examine: (i) the anatomical morphology of the fabella; and (ii) the relationship between the classification of fabellae and the severity of KOA.

## Patients and Methods

### Ethical Statement

All the procedures were approved by the Ethics Committee of at Affiliated Traditional Chinese Medicine Hospital of Southwest Medical University (No. KY2018030) and all methods were performed in accordance with the relevant guidelines and regulations. All the measurements of fabella and KOA were collected at the Radiology Department of Affiliated Traditional Chinese Medicine Hospital of Southwest Medical University (Luzhou, China).

### Instruments

KOA was measured by Magnetic resonance imaging (MAGNETON; Skyra, 3.0T) and these images were stored in the Picture Archiving Communication System (PACS; DJ Health Union Systems Corporation, Shanghai, China). After computed tomography (CT) scanning (Somatom Emotion; Siemens AG, Munich, Germany), the images of fabella were reconstructed in 3D by syngoMMWP VE40B and all 3D images were stored in the Picture Archiving Communication System. PACS (UniReport version 2.0) can record and store a large number of images and assist in accurate measuring.

### Inclusion and Exclusion Criteria

Inclusion criteria: (i) diagnosed with KOA, according to the criteria of the American College of Rheumatology; (ii) patient scans of fabella and KOA must be clear and intact, and the basic information and imaging data complete; (iii) outcome measures are Recht grade, short axis, long axis, the distance between two fabellae, and the distance between the proximal section of the femoral condyle and the section of the fabella; (iv) retrospective study. Exclusion criteria: (i) previous knee injury or joint infection, such as patients with a history of systemic, rheumatic, or inflammatory disease or chondrocalcinosis, hemochromatosis, inflammatory arthritis; (ii) patients who had contraindications for 3.0T magnetic resonance imaging (MRI) or CT.

### Patients

A total of 302 patients who had KOA detected on 3.0T MRI at the Affiliated Traditional Chinese Medicine Hospital of Southwest Medical University were considered for the study. Informed consent was obtained from all subjects. But after measuring by a spiral CT scanner, 136 patients who had fabella, KOA, and met inclusion and exclusion criteria were included. They included 68 left sides and 68 right sides, 51 males and 85 females (mean age 62.71 ± 10.75 years).

### Measurements

**Fig. 1:**
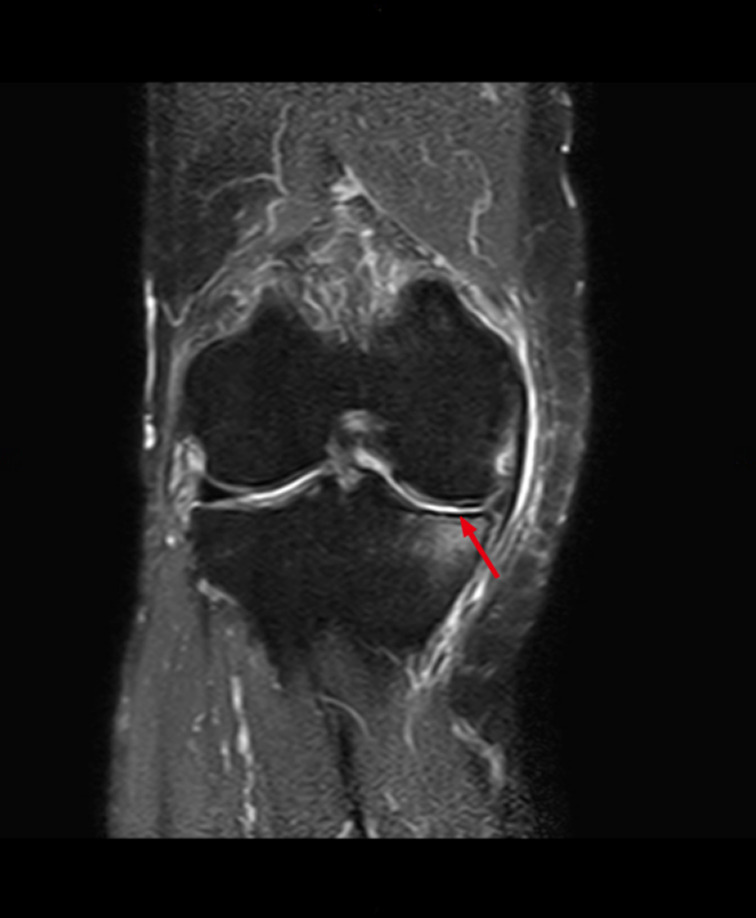
Measurement of the severity of KOA. Coronal intermediate‐weighted fat suppressed MRI shows focal cartilage damage (red arrow).

After acquiring the 3D reconstruction models of the fabella and MRI image of KOA, the measurement was made by two researchers who had engaged in radiology work for more than 3 years. When there was a divergence, a third observer eventually decided. These researchers would take measurements all alone and each measurement was repeated three times, before averaging the three values. The severity of KOA was assessed by Recht grade^26^ (grade 0, normal cartilage; grade I, cartilage softening and/or swelling; grade II, mild surface fibrillation and/or less than 50% loss of cartilage thickness; grade III, severe surface fibrillation and/or loss of more than 50% of cartilage thickness but without exposure of subchondral bone; and grade IV, complete loss of cartilage with subchondral bone exposure) (Fig. 1).

**Fig. 2:**
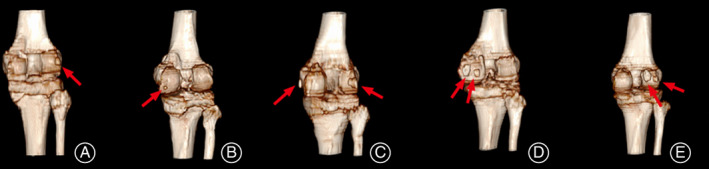
Various types of the fabellae. The fabella showed by red arrow. (A) A fabella on the lateral femoral condyle. (B) A fabella on the medial femoral condyle. (C) A fabella on the lateral femoral condyle and a fabella on the medial femoral condyle. (D) Two fabellae on the medial femoral condyle. (E) Two fabellae on the lateral femoral condyle.

According to CT imaging characteristics, the fabella was classified into five types based on the position and quantity (Fig. 2).

Type I: A fabella on the lateral femoral condyle.

Type II: A fabella on the medial femoral condyle.

Type III: A fabella on the lateral femoral condyle and a fabella on the medial femoral condyle.

Type IV: Two fabellae on the medial femoral condyle.

Type V: Two fabellae on the lateral femoral condyle.

The following parameters were defined and measured (accurate to 0.01 cm) in the 3D reconstruction models.

Short axis: The short axis of fabella. (The fabellae with two were determined by calculating an average value).

Long axis: The long axis of fabella. (The fabellae with two were determined by calculating an average value).

**Fig. 3:**
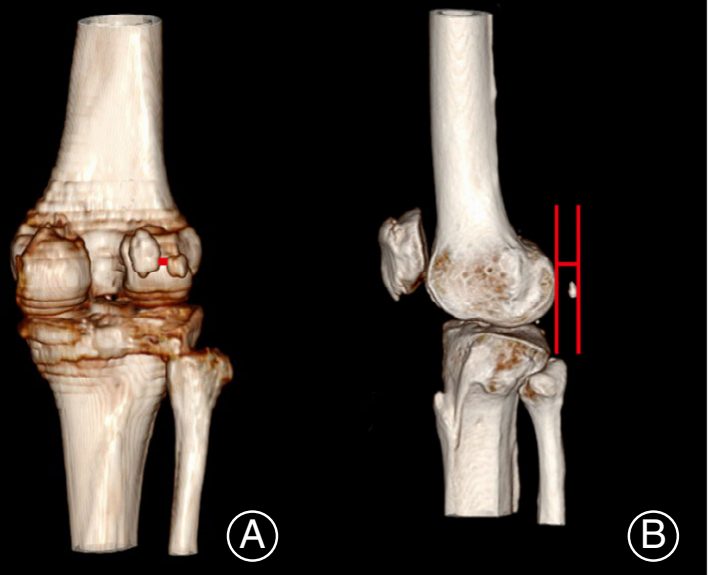
Measurement of the fabella. (A) The distance between two fabellae (red line). (B) The distance between the proximal section of the femoral condyle and the section of the fabella (red line).

A: The distance between two fabellae (Fig. 3).

B: The distance between the proximal section of the femoral condyle and the section of the fabella (Fig. 3).

### Statistical Analysis

Statistical analysis was performed by using SPSS version 24.0 (IBM Corp, Armonk, NY, USA). All data were presented by the mean ± standard deviation (SD). Categorical variables were recorded as numbers and percentages with frequency tables. The significance level was set at *P* = 0.05. One‐way ANOVA, non‐parametric tests, and the Shapiro–Wilk test were applied to analyze differences about the anatomic parameters of the fabella and classification. The differences in the fabellar classification and the severity of KOA was assessed using Conover W. J. test. The Spearman nonparametric correlation test was used for correlative analysis.

## Results

### Classification of Fabellae

According to the location and quantity of fabellae, the fabellae were divided into five types: type I (71, 52.21%), type II (44, 32.35%), type III (nine, 6.62%), type IV (four, 2.94%), and type V (eight, 5.88%). Among these classifications, type I was the most common while type IV was the lowest. The short axis of type III (0.59 ± 0.28 cm) was significantly larger than type I (0.45 ± 0.19 cm) and type II (0.45 ± 0.18 cm), and the difference was statistically significant (*P* < 0.05). With regard to the long axis, type IV (1.21 ± 0.76 cm) was significantly larger than other types, except for type III (*P* < 0.05). Type III (1.04 ± 0.41 cm) was larger than type I (0.80 ± 0.26 cm) and type II (0.80 ± 0.35 cm), and there was significant difference (*P* < 0.05). In term of A and B, there were no significant statistical differences between different types (*P* > 0.05). The results are displayed on Table 1.

**TABLE 1:** The measurement of the fabella based on classification (Mean ± SD)

	Type I	Type II	Type III	Type IV	Type V	Total
Number	71	44	9	4	8	136
Ratio	52.21%	32.35%	6.62%	2.94%	5.88%	100%
Short axis (cm)	0.45 ± 0.19	0.45 ± 0.18	0.59 ± 0.28^*^ ^†^	0.52 ± 0.26	0.52 ± 0.26	0.48 ± 0.21
Long axis (cm)	0.80 ± 0.26	0.80 ± 0.35	1.04 ± 0.41^*^ ^†^	1.21 ± 0.76^*^ ^†^	0.86 ± 0.50^‡^	0.86 ± 0.38
A(cm)	‐	‐	3.82 ± 1.85	1.34 ± 0.26	1.03 ± 0.56	2.28 ± 1.83
B(cm)	0.54 ± 0.54	0.63 ± 0.54	0.62 ± 0.42	0.59 ± 0.41	0.61 ± 0.68	0.58 ± 0.53

A: The distance between two fabellae. B: The distance between the proximal section of the femoral condyle and the section of the fabella.

^*^ *P* < 0.05 *vs* Type I.

^†^ *P* < 0.05 *vs* Type II.

^‡^ *P* < 0.05 *vs* Type IV.

### KOA Grades

The classification of fabellae were correlated with KOA grades (χ^2^ = 35.026, *P* < 0.05). In terms of KOA grades, grade I and grade II occupied most of type II (32, 72.8%), type II and other types showed significant statistical difference (*P* < 0.05). Grade I and grade II were mainly type IV (four, 100%). Type V's biggest component was grade III and grade IV (six, 75%). Type IV and type V showed significant statistical difference (*P* < 0.05). The results are displayed on Table 2.

**TABLE 2:** The interrelation between classification of fabellae and the severity of KOA

Fabella			KOA		Total
	Grade I	Grade II	Grade III	Grade IV	
Type I number	4	16	25	26	71
Ratio	5.6%	22.5%	35.2%	36.6%	100.0%
Type II number	9	23	10	2	44^*^
Ratio	20.5%	52.3%	22.7%	4.5%	100.0%
Type III number	1	1	3	4	9^†^
Ratio	11.1%	11.1%	33.3%	44.4%	100.0%
Type IV number	1	3	0	0	4^*^ ^‡^
Ratio	25.0%	75.0%	0%	0%	100.0%
Type V number	1	1	2	4	8^†^ ^§^
Ratio	12.5%	12.5%	25.0%	50.0%	100.0%
Total number	16	44	40	36	136
Ratio	11.8%	32.4%	29.4%	26.5%	100.0%

^*^ *P* < 0.05 *vs* Type I.

^†^ *P* < 0.05 *vs* Type II.

^‡^ *P* < 0.05 *vs* Type III.

^§^ *P* < 0.05 *vs* Type IV.

## Discussion

Conventional radiography of the Kellgren–Lawrence stage division has been considered as a standard for describing the severity of KOA^27^. However, we choose the MRI of Recht grade as a result of it being able to assess soft tissue and KOA affects in the articular cartilage^28^, ^29^. The primary approach currently available for KOA diagnosis is MRI, which aids in diagnosing KOA, determining KOA progression and prognosis, and monitoring treatment responses^30^. Using radiography alone to measure the loss of cartilage has limited clinical utility and only a modest correlation with symptom severity. Instead, MRI has consistently been seen to have the capacity to be predictive of KOA symptoms^31^. Various studies demonstrated that MRI is highly specific and moderately sensitive and accurate for identifying articular cartilage degeneration of any severity, so it has become an essential research tool for KOA studies^32^, ^33^, ^34^.

The mean age of KOA is 62.71 ± 10.75 years and there are 51 males and 85 females in this study; this is consistent with what the published articles have reported, that women have a higher prevalence of KOA and KOA primarily affects the elderly population worldwide^35^, ^36^. Among these classifications, type I was the most common. The average range for short axis and long axis is 0.48 ± 0.21 cm and 0.86 ± 0.38 cm, respectively. But some studies reported the fabella usually ranges from 0.5 to 2 cm in diameter in the Chinese population. We hypothesized that this difference may be based on race^37^, ^38^. The short axis of type III (0.59 ± 0.28 cm) was significantly larger than type I and type II (*P* < 0.05). Concerning the long axis, type IV (1.21 ± 0.76 cm) was significantly larger than other types, except for type III (*P* < 0.05). Type III (1.04 ± 0.41 cm) was larger than type I and type II (*P* < 0.05). These results showed that the variability of the long and short axis between different types means that we should pay attention to this difference when fabella‐related illness occurs. In terms of the distance between the proximal section of the femoral condyle and the section of the fabella, there was no significant statistical differences between different types (*P* > 0.05). This demonstrated that the difference of B (The distance between the proximal section of the femoral condyle and the section of the fabella) is very little between different types and might be useful for localizing the fabella and scheduling the arthroscopic and surgical approach.

The treatment of fabella‐related illness includes physical therapy, injection of local anesthetics or steroids around this bone, radial extracorporeal shock wave therapy or fabellectomy^39^. As fabella could cause KOA, it may be an atavistic pattern. Some people insisted that fabellae could be excised and found the posterolateral pain would disappear or greatly improve when removing the fabella^40^. Type V has corresponded to the higher grade of KOA. So, we speculate that if the imaging performance of fabella indicates type V, we could predispose the fabella to prevent the occurrence and progression of KOA.

This study had some limitations. First, as the prevalence of type III, IV, and V was too low, this study's sample capacity was relatively limited, which would cause sampling bias. Second, to provide a personalized treatment of KOA, further studies on the relationship between different classifications of fabellar and the severity of KOA are encouraged.

## Conclusion

According to the location and quantity of fabellae, the fabella was divided into five types and type I was the most common. The classification of fabellae were correlated with KOA grades. Type II may mean lower KOA grades, while type V may mean higher KOA grades.

## Declarations

### Ethics Approval

All the procedures were approved by the Ethics Inspection Committee at Affiliated Traditional Chinese Medicine Hospital of Southwest Medical University (No. KY2018030). All patients signed a General Consent of the Ethical Committee of the Affiliated Traditional Chinese Medicine Hospital of Southwest Medical University for using and publishing their data for scientific use.

### Consent to Participate

All the patients agreed to participate.

### Consent for Publication

All the authors agreed to publish.

### Availability of Data and Materials

The datasets generated during and/or which were analyzed during the current study are available from the corresponding author on reasonable request.

### Code Availability

The software application is available from the corresponding author on reasonable request.

### Authors' Contributions

LZ and GYW contributed to conception and design of study. YLW and CYH contributed to writing and editing this manuscript. YZ contributed to protocol and project development of study. JQW contributed to data collection and literature search. All authors read and approved the final manuscript.
